# Two-Dimensional Perovskite Crystals Formed by Atomic Layer Deposition of CaTiO_3_ on γ-Al_2_O_3_

**DOI:** 10.3390/nano11092207

**Published:** 2021-08-27

**Authors:** Tianyu Cao, Ohhun Kwon, Chao Lin, John M. Vohs, Raymond J. Gorte

**Affiliations:** Department of Chemical and Biomolecular Engineering, University of Pennsylvania, Philadelphia, PA 19104, USA; caot@seas.upenn.edu (T.C.); ohhun@seas.upenn.edu (O.K.); linchao@seas.upenn.edu (C.L.); vohs@seas.upenn.edu (J.M.V.)

**Keywords:** perovskites, thin films, crystallization, atomic layer deposition

## Abstract

CaTiO_3_ films with an average thickness of 0.5 nm were deposited onto γ-Al_2_O_3_ by Atomic Layer Deposition (ALD) and then characterized by a range of techniques, including X-ray Diffraction (XRD) and High-Resolution, Transmission Electron Microscopy (HRTEM). The results demonstrate that the films form two-dimensional crystallites over the entire surface. Lattice fringes from HRTEM indicate that the crystallites range in size from 5 to 20 nm and are oriented in various directions. Films of the same thickness on SiO_2_ remained amorphous, indicating that the support played a role in forming the crystallites.

## 1. Introduction

Thin oxide films on high-surface-area substrates could be important for a number of applications, including novel heterogeneous catalysts [1] and new materials for chemical looping [2]. The standard impregnation of metal salts can give monolayer oxides when there are favorable interactions with the support, such as in the case of titania-supported vanadia catalysts [3]; however, when the impregnated amount is in excess of a monolayer, three-dimensional particles are usually formed [4]. Recent interest in adding the second oxide by Atomic Layer Deposition (ALD) results from the fact that uniform, two-dimensional layers which are thicker than one monolayer can be formed by this procedure [5,6,7,8]. In some applications, the crystallinity of the two-dimensional film could be very important.

An interesting example where thin films are important is that of perovskite-supported metals. These have been referred to as “Intelligent Catalysts” because the reversible ingress and egress of the metal catalyst into the perovskite lattice can potentially be used to redisperse the metal after sintering [9,10]. Strong interactions between the perovskite and the metal can also affect other properties of the metal. For example, Ni supported on Ti-based perovskites have been shown to exhibit extreme tolerance against coking [11] while still showing a high activity for the reforming of methane [12]. However, the implementation of these catalysts has been limited by the low surface areas of typical perovskites and by the fact that much of the metal remains in the perovskite lattice, inaccessible for reactions [1]. Both problems can be solved by having the perovskite in the form of a thin film on a second, high-surface-area oxide. Since the perovskite structure of the oxide is critical for establishing the catalytic properties of Intelligent Catalysts [13], the characterization of the structure of the film is critical.

For a supported thin film to retain a high surface area, the thickness cannot be greater than about 1 nm. This is illustrated by considering that a 1 nm film with a density of 5 g/cm^3^ on a 200 m^2^/g support would have a mass equal to that of the underlying support. Assuming that the surface area does not decrease even more due to the shrinkage of the pores, the specific surface area would still decrease by a factor of two simply due to the increase in the mass of the sample. However, since the unit cell size of oxides with perovskite or fluorite structures is only about 0.5 nm, the crystallinity of a 1 nm film would depend on the in-plane ordering of the cations. The characterization of this ordering by techniques like X-ray Diffraction (XRD) is challenging since the film thickness is less than the X-ray coherence length.

Consistent with the films being thinner than the X-ray coherence length, studies from our laboratories of CeO_2_ [14], ZrO_2_ [15], and CeZrO_4_ [16] films less than 1 nm in thickness and deposited onto Al_2_O_3_ by ALD showed these to be X-ray amorphous, even for oxide loadings as high as nearly 40 wt% and calcination temperatures above 1073 K. However, later investigations of mixed oxides with stoichiometries of LaFeO_3_ [17,18] and CaTiO_3_ [12,19] reported intense perovskite XRD peaks for even lower loadings on MgAl_2_O_4_ supports, even though other characterization techniques indicated that the films remained intact and did not coalesce into particles. Furthermore, X-ray line broadening of the perovskite features indicated crystallite sizes between 10 and 20 nm [1], which was inconsistent with there being sufficient material to cover the surface of the support. Since diffraction in the direction perpendicular to the plane of a two-dimensional crystallite is possible [20], it was suggested that the films formed flat crystallites with random surface orientations [1]. The direct observation of these crystallites by electron microscopy was difficult however, due to the underlying crystallinity of the MgAl_2_O_4_ support.

In the present study, we investigated CaTiO_3_ films on a γ-Al_2_O_3_ support to look for direct evidence of crystallinity in two-dimensional films. In previous research, CaTiO_3_ film has proven itself a promising support for both noble metal catalysts [19,21] and Ni-based catalysts [12]. γ-Al_2_O_3_ was chosen as the support because of its relatively poor crystallinity and low reactivity with CaTiO_3_. Although CaAl_2_O_4_ formation is possible, the spinel structure is easily distinguished from that of a perovskite phase if it should form.

## 2. Materials and Methods

### 2.1. Sample Preparation

Fresh γ-Al_2_O_3_ (Strem Chemicals, Inc., Newburyport, MA, USA, 180 m^2^/g) was first calcined in air at 1173 K for 24 h to achieve a stable support with a surface area of 105 m^2^/g, and the diameter of the calcined Al_2_O_3_ particles ranged between 5–30 nm. ALD was performed in a static system that has been described in detail elsewhere [15]. The precursors were Ca(TMHD)_2_ (TMHD=2,2,6,6-tetramethyl-3,5-heptanedionato, Strem Chemicals, Inc., Newburyport, MA, USA) and TiCl_4_ (Sigma-Aldrich, St. Louis, MO, USA). For CaO deposition, the sample was exposed to vapors from the Ca precursor for 5 min at 573 K; ligand removal was accomplished by oxidizing the sample in air at 873 K in a muffle oven for 5 min. For TiO_2_ deposition, the sample was exposed to TiCl_4_ vapor at 423 K for 3 min; oxidation was accomplished by exposure to humidified air (containing 10% steam) at 423 K. Growth rates were determined by weighing the sample after every ALD cycle, as shown in Figure S3, and were found to be 6.6 × 10^13^ Ca atom/cm^2^-cycle (equivalent to 0.018 nm of CaO per cycle, assuming a bulk density for the film) and 9.6 × 10^13^ Ti atom/cm^2^-cycle (0.030 nm of TiO_2_ per cycle). To achieve the correct perovskite stoichiometry, we performed six ALD cycles of CaO, followed by four cycles of TiO_2_. The elemental composition of the deposited sample was further tested by the inductively coupled plasma−optical emission spectrometry (ICP-OES) method on a Spectro Genesis spectrometer equipped with a Mod Lichte nebulizer. Finally, the CaTiO_3_/Al_2_O_3_ sample was calcined in air at 1073 K for 3 h. The calculated thickness of the CaTiO_3_ film was 0.52 nm, determined from the mass of deposited CaTiO_3_ and the BET surface area of the Al_2_O_3_, assuming a uniform film with the bulk density of CaTiO_3_ (3.98 g cm^−3^) [12].

### 2.2. Sample Characterization

X-ray Diffraction (XRD) was performed on a Rigaku MiniFlex diffractometer (Rigaku Analytical Devices, Inc., Wilmington, MA, USA) equipped with a Cu K-α source (λ = 0.15406 nm). Scanning transmission electron microscopy (STEM) and high-resolution transmission electron microscopy (HR-TEM) images and Energy Dispersive X-ray Spectra (EDS) were acquired on a JEOL JEM-F200 STEM (JEOL USA Inc., Peabody, MA, USA), operated at 200 kV. For the TEM analysis, the sample was ground with a mortar and pestle and then dispersed in ethanol to form a suspension. A single drop of the suspension was then added to a standard TEM sample grid (Ted Pella, Inc., Redding, CA, USA), which was then allowed to dry. Temperature Programmed Desorption (TPD) measurements were performed in high vacuum using equipment that is described in detail elsewhere [22]. During the TPD experiment, the samples were first exposed to 2-propanol vapor at room temperature, then evacuated for 1 h using a turbo-molecular pump. The sample temperature was then ramped at 10 K min^−1^ while monitoring the desorbing species using a quadrupole mass spectrometer (SRS-RGA-100, Stanford Research Systems, Sunnyvale, CA, USA).

## 3. Results

**Figure 1:**
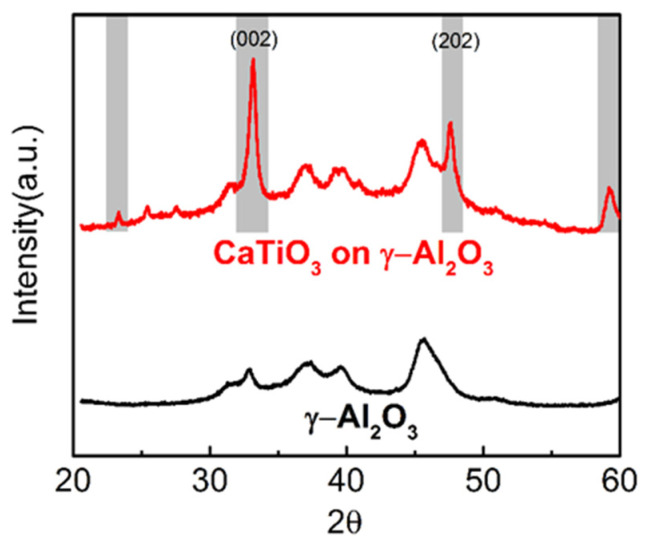
XRD patterns of bare Al_2_O_3_ (γ-phase, denoted as the black line) and the 18 wt% CaTiO_3_/Al2O_3_ sample (denoted as the red line). Features shaded in grey correspond to peaks of the perovskite phase.

The XRD patterns of the 18 wt% CaTiO_3_/Al_2_O_3_ sample and the bare Al_2_O_3_ support are presented in Figure 1. The pattern for bare Al_2_O_3_ shows weak, broad peaks, consistent with a low degree of crystallinity, while the CaTiO_3_-containing sample shows additional features at 23, 33, 48, 59 degrees 2θ that can be assigned to the CaTiO_3_ perovskite phase, even though the thickness of the film (0.52 nm) was much smaller than the coherence length of XRD. Based on the linewidth of the feature at ~33 degrees, the crystallite size of the perovskite phase is estimated to be ~17 nm. The two small peaks at 25 and 27 degrees can be assigned to the (101) peak of an anatase phase and the (110) peak of a rutile phase. Both peaks are the strongest features of their respective phases and suggest that there was a slight excess of TiO_2_ in the sample. The ICP-OES results showed that the weight loading of CaTiO_3_ on Al_2_O_3_ was 16.7 wt%, consistent with that acquired by weight tracking. The ratio between Ca and Ti was 0.95:1 and agreed with the XRD results. It is worth noting that if all of the perovskite phase were present as three-dimensional particles, 17 nm in size, the amount of CaTiO_3_ in the sample would be sufficient to cover only a very small fraction of the Al_2_O_3_.

**Figure 2:**
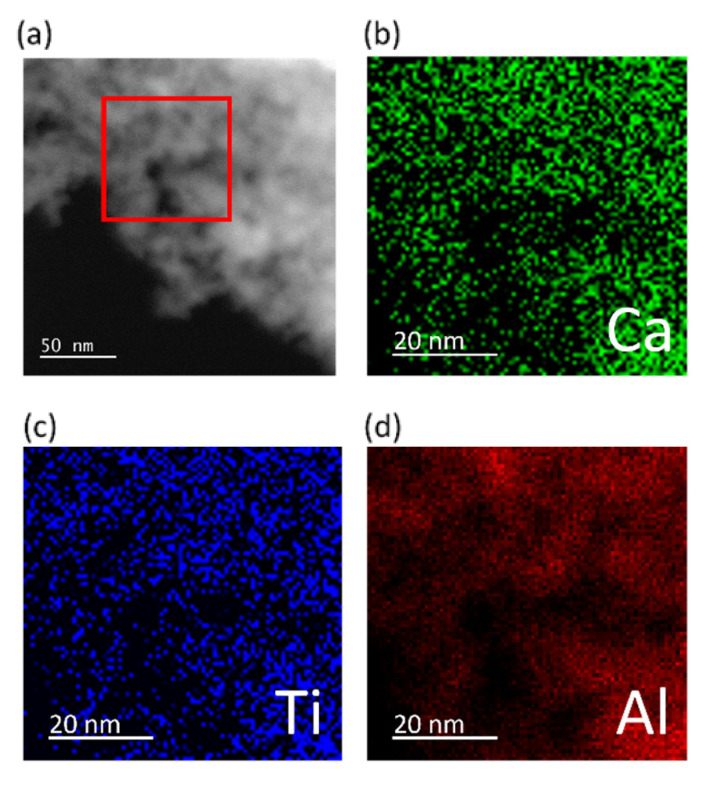
(**a**) High angle annular dark field (HAADF) STEM image of the 18 wt% CaTiO_3_/Al_2_O_3_ sample; (**b**–**d**) are EDS maps of Ca, Ti and Al, taken from the region indicated by the red frame.

To determine the morphology of the added CaTiO_3_, Scanning Transmission Electron Microscope (STEM) images with Energy Dispersive X-ray Spectra (EDS) were obtained, as shown in Figure 2a–d. The STEM images of CaTiO_3_/Al_2_O_3_ were indistinguishable from those of Al_2_O_3_. The EDS maps of Ca, Ti and Al were more informative and show uniform distributions of Ca and Ti over the entire surface.

**Figure 3:**
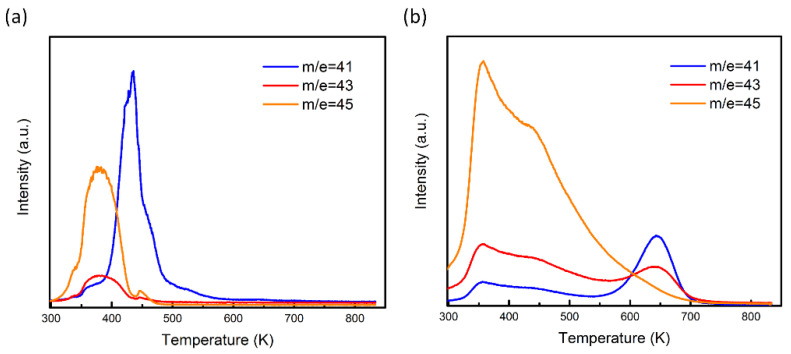
TPD of 2-propanol from (**a**) bare Al_2_O_3_; (**b**) Al_2_O_3_ loaded with 18 wt% CaTiO_3_. Desorption features correspond to propene (m/e = 41), acetone (m/e = 43), and unreacted 2-propanol (m/e = 41, 43, and 45).

Further evidence for the CaTiO_3_ forming a uniform film comes from Temperature Programmed Desorption (TPD) measurements reported in Figure 3. Al_2_O_3_ is a Lewis acid and is a good catalyst for alcohol dehydration. As discussed in more detail elsewhere [23], room-temperature exposure of Al_2_O_3_ to 2-propanol, followed by evacuation, leaves approximately one monolayer of the alcohol on the surface. As shown by the TPD in Figure 3a, about half of this adsorbed alcohol leaves the surface as 2-propanol (m/e = 45, 43, and 41) below 400 K, with the rest reacting to propene (m/e = 41) and water, with propene desorbing between 400 and 500 K. The analogous results for the 18 wt% CaTiO_3_/Al_2_O_3_ sample are presented in Figure 3b. Significant amounts of unreacted 2-propanol again desorbed from the sample below 400 K. However, we also observed acetone (m/e = 43) and propene from 575 K to 700 K. For the present purposes, it is noteworthy that desorption features associated with the bare Al_2_O_3_ are completely absent. If a significant fraction of the Al_2_O_3_ remained uncovered, one would have observed a propene feature in the TPD below 500 K.

**Figure 4:**
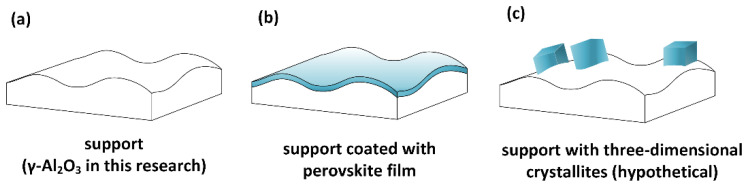
Morphology of the CaTiO_3_ film on the surface of the Al_2_O_3_ support. (**a**) Bare support; (**b**) support coated with a uniform film; (**c**) support decorated with three-dimensional cubic crystallites.

While the XRD pattern suggested that the CaTiO_3_ was present in the form of ~17 nm crystallites, the STEM results in Figure 2 and the TPD results in Figure 3 demonstrated that the CaTiO_3_ must be present as a uniform film no more than ~0.5 nm in thickness. However, if even a fraction of the 17 nm crystallites were three-dimensional, there would be insufficient material to completely cover the Al_2_O_3_ support. This is shown diagrammatically in Figure 4, in which Figure 4a demonstrates the pristine state of the Al_2_O_3_ support. If the crystallized CaTiO_3_ were present as three-dimensional, cubic crystallites with an edge length of 10 nm shown in Figure 4c, then 18 wt% CaTiO_3_ would be sufficient to occupy only 5% of the 105 m^2^/g Al_2_O_3_. Both the STEM/EDS and 2-propanol TPD results imply that the morphology of the film must be more similar to that shown in Figure 4b.

**Figure 5:**
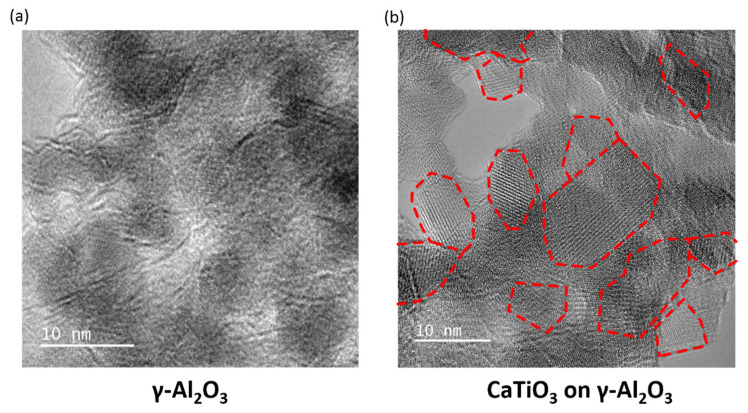
HR-TEM image of (**a**) bare Al_2_O_3_; (**b**) 18 wt% CaTiO_3_/Al_2_O_3_.

The crystallinity of the CaTiO_3_ films was further assessed by High Resolution Transmission Electron Microscopy (HR-TEM), with representative images of the bare and CaTiO_3_-covered Al_2_O_3_ reported in Figure 5. The image of Al_2_O_3_ in Figure 5a does not exhibit any well-defined lattice fringes, consistent with the broad peaks found in the XRD pattern of Figure 1. By contrast, the entire surface of the CaTiO_3_/Al_2_O_3_ sample, shown in Figure 5b, is covered with lattice fringes. Distinguishable crystallites in Figure 5b are framed by the dashed red lines. Most of the crystallites seem to be between 5 and 10 nm in size, with some of the fringes extending over significantly larger dimensions.

**Figure 6:**
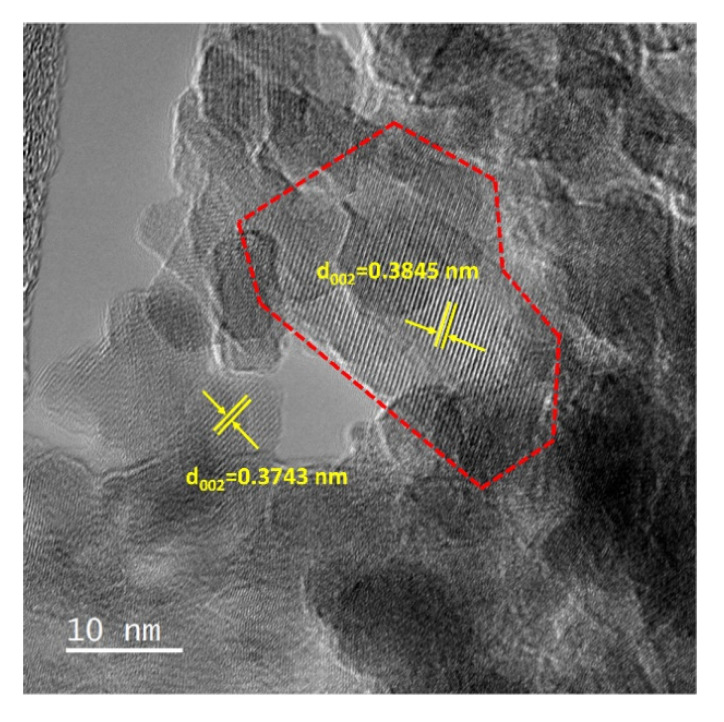
HR-TEM image of the 18 wt% CaTiO_3_/Al_2_O_3_ sample.

In the HR-TEM image of the CaTiO_3_/Al_2_O_3_ sample in Figure 6, we focused on regions of the sample that exhibited better crystallization in order to index some of the observed planes. As shown in yellow, fringes could be easily identified with d-spacing corresponding to (002) planes, consistent with these being the most intense feature in the XRD patterns presented in Figure 1. At least one of these crystallites appears to be greater than 20 nm in size (denoted as the red-framed region in Figure 6), consistent with the crystallite-size estimates from XRD. Lattice spacings corresponding to the (202) planes were observed in other images, as shown in Figure S1 (The lattice parameters as well as the d-spacing values for bulk CaTiO_3_ are presented in Table S1).

The question arises whether the Al_2_O_3_ support plays a role in crystallizing the perovskite film. To address this issue, we also deposited a 0.5 nm film (0.37 g/g SiO_2_) of a mixed oxide with the CaTiO_3_ stoichiometry (The Ca:Ti ratio of the sample was 0.93:1 based on the ICP-OES results) onto an amorphous silica that had a surface area of 180 m^2^/g. Growth rates of CaO and TiO_2_ on SiO_2_ are presented in Figure S4. The sample was again oxidized at 1073 K for 3 h. As shown in Figure S2, the film was largely amorphous, except for a few minor peaks corresponding to an anatase phase (TiO_2_). The absence of diffraction peaks showed conclusively that three-dimensional particles were not formed and that the film was also not crystalline on the length scale of the X-ray. This result is similar to what was observed in earlier attempts to prepare LaFeO_3_ films on silica, which also failed to find evidence of a perovskite phase from diffraction measurements [24].

## 4. Discussion

It is interesting to ask why the results on SiO_2_ are so different from those on Al_2_O_3_ and MgAl_2_O_4_ [21,25]. Clearly, the perovskite films are not epitaxially matched to the crystal structures of Al_2_O_3_ (cubic, a = 0.7912 nm) or MgAl_2_O_4_ (cubic, a = 0.8081 nm) [12]. However, it appears that both Al_2_O_3_ and MgAl_2_O_4_ are able to nucleate the formation of larger, two-dimensional crystallites while SiO_2_ is not. Because the crystallization of a perovskite thin film is a nucleation-controlled process, understanding the manner of nucleation is important. Given the thickness of the thin film in the present research (~0.5 nm), the perovskite film must have crystallized in a “heterogeneous nucleation” manner, during which the “seeding effect” played a major role [26]. The nanoscale seeds at the interface between the overlayer and its support presumably reduce the energy barrier for forming crystallites by promoting nucleation at a specific surface site, thus lowering the temperature required to crystallize the perovskite phase. Seeds that were previously reported to facilitate nucleation are crystallized materials, such as perovskite [27] or an intermetal [28]. In the present study, surface crystallites of Al_2_O_3_ likely served as the seeds in the nucleation of CaTiO_3_. By contrast, the crystallization of a perovskite phase on the surface of SiO_2_ is more difficult due to a scarcity of nucleation sites. A better understanding of the crystallization process could be achieved with theoretical tools such as the density function theory (DFT).

The diffraction pattern for CaTiO_3_/Al_2_O_3_ exhibits the typical powder-pattern behavior, and a better understanding of the nucleation process may provide an opportunity to prepare materials in which certain crystal planes are preferred.

## 5. Conclusions

We demonstrated that relatively large, two-dimensional perovskite platelets were formed when CaTiO_3_ films were deposited onto γ-Al_2_O_3_ by ALD. The Al_2_O_3_ support facilitated the crystallization of its perovskite overlayer by providing sites for nucleation. The formation of CaTiO_3_ crystallites on the amorphous SiO_2_ support, however, is more difficult.

## Data Availability

The data presented in this study are available upon request from the corresponding author.

## Supplementary Materials

The following are available online at https://www.mdpi.com/article/10.3390/nano11092207/s1, Figure S1: (a) High angle annular dark field (HAADF) STEM image of the 18-wt% CaTiO_3_/Al_2_O_3_ sample; (b–d) are EDS maps of Ca, Ti and Al, taken from the region indicated by the dashed red frame; (e) HR-TEM of the sample, image acquired from the orange framed region, Figure S2: XRD patterns of CaTiO_3_ deposited on SiO_2_, the black line denotes bare SiO_2_ and the red line denotes CaTiO_3_/SiO_2_, Figure S3: Growth rate of CaO, TiO_2_ on γ-Al_2_O_3_ as a function of ALD cycles. (a) CaO; (b) TiO_2_, Figure S4: Growth rate of CaO, TiO_2_ on SiO_2_ as a function of ALD cycles. (a) CaO; (b) TiO_2_, Table S1: Lattice perimeters and d-spacing values of standard CaTiO_3_ crystal system.

## References

[1] Mao X., Lin C., Graham G.W., Gorte R.J. (2020). A Perspective on Thin-Film Perovskites as Supports for Metal Catalysts. ACS Catal..

[2] Scheffe J.R., Allendorf M.D., Coker E.N., Jacobs B.W., McDaniel A.H., Weimer A.W. (2011). Hydrogen production via chemical looping redox cycles using atomic layer deposition-synthesized iron oxide and cobalt ferrites. Chem. Mater..

[3] Deo G., Wachs I.E. (1991). Predicting molecular structures of surface metal oxide species on oxide supports under ambient conditions. J. Phys. Chem..

[4] Su S.C., Bell A.T. (1998). A study of the structure of vanadium oxide dispersed on zirconia. J. Phys. Chem. B.

[5] Canlas C.P., Lu J., Ray N.A., Grosso-Giordano N.A., Lee S., Elam J.W., Winans R.E., Van Duyne R.P., Stair P.C., Notestein J.M. (2012). Shape-selective sieving layers on an oxide catalyst surface. Nat. Chem..

[6] O’Neill B.J., Jackson D.H., Lee J., Canlas C., Stair P.C., Marshall C.L., Elam J.W., Kuech T.F., Dumesic J.A., Huber G.W. (2015). Catalyst design with atomic layer deposition. ACS Catal..

[7] Jackson D.H., Kuech T.F. (2017). Electrochemical effects of annealing on atomic layer deposited Al_2_O_3_ coatings on LiNi_0.5_Mn_0.3_Co_0.2_O_2_. J. Power Sources.

[8] Singh J.A., Yang N., Bent S.F. (2017). Nanoengineering heterogeneous catalysts by atomic layer deposition. Annu. Rev. Chem. Biomol. Eng..

[9] Nishihata Y., Mizuki J., Akao T., Tanaka H., Uenishi M., Kimura M., Okamoto T., Hamada N. (2002). Self-regeneration of a Pd-perovskite catalyst for automotive emissions control. Nature.

[10] Tanaka H., Tan I., Uenishi M., Kimura M., Dohmae K. (2001). Regeneration of palladium subsequent to solid solution and segregation in a perovskite catalyst: An intelligent catalyst. Top. Catal..

[11] Neagu D., Oh T.-S., Miller D.N., Ménard H., Bukhari S.M., Gamble S.R., Gorte R.J., Vohs J.M., Irvine J.T. (2015). Nano-socketed nickel particles with enhanced coking resistance grown in situ by redox exsolution. Nat. Commun..

[12] Lin C., Jang J.B., Zhang L., Stach E.A., Gorte R.J. (2018). Improved Coking Resistance of “Intelligent” Ni Catalysts Prepared by Atomic Layer Deposition. ACS Catal..

[13] Mao X., Foucher A.C., Stach E.A., Gorte R.J. (2019). “Intelligent” Pt Catalysts Based on Thin LaCoO_3_ Films Prepared by Atomic Layer Deposition. Inorganics.

[14] Onn T.M., Zhang S., Arroyo-Ramirez L., Xia Y., Wang C., Pan X., Graham G.W., Gorte R.J. (2017). High-surface-area ceria prepared by ALD on Al_2_O_3_ support. Appl. Catal. B Environ..

[15] Onn T.M., Zhang S., Arroyo-Ramirez L., Chung Y.-C., Graham G.W., Pan X., Gorte R.J. (2015). Improved thermal stability and methane-oxidation activity of Pd/Al_2_O_3_ catalysts by atomic layer deposition of ZrO_2_. ACS Catal..

[16] Onn T.M., Dai S., Chen J., Pan X., Graham G.W., Gorte R.J. (2017). High-Surface Area Ceria-Zirconia Films Prepared by Atomic Layer Deposition. Catal. Lett..

[17] Mao X., Foucher A.C., Montini T., Stach E.A., Fornasiero P., Gorte R.J. (2020). Epitaxial and Strong Support Interactions between Pt and LaFeO_3_ Films Stabilize Pt Dispersion. J. Am. Chem. Soc..

[18] Mao X., Foucher A.C., Stach E.A., Gorte R.J. (2020). Changes in Ni-NiO equilibrium due to LaFeO_3_ and the effect on dry reforming of CH_4_. J. Catal..

[19] Lin C., Foucher A.C., Ji Y., Curran C.D., Stach E.A., McIntosh S., Gorte R.J. (2019). “Intelligent” Pt Catalysts Studied on High-Surface-Area CaTiO_3_ Films. ACS Catal..

[20] Holder C.F., Schaak R.E. (2019). Tutorial on Powder X-ray Diffraction for Characterizing Nanoscale Materials. ACS Nano.

[21] Lin C., Foucher A., Ji Y., Stach E.A., Gorte R.J. (2020). Investigation of Rh-titanate (ATiO_3_) interactions on high-surface-area perovskites thin films prepared by atomic layer deposition. J. Mater. Chem. A.

[22] Cao T., Huang R., Gorte R.J., Vohs J.M. (2020). Endothermic reactions of 1-propanamine on a zirconia catalyst. Appl. Catal. A Gen..

[23] Roy S., Mpourmpakis G., Hong D.-Y., Vlachos D.G., Bhan A., Gorte R. (2012). Mechanistic study of alcohol dehydration on γ-Al_2_O_3_. ACS Catal..

[24] Cao T., Kwon O., Vohs J.M., Gorte R.J. (2021). LaFeO_3_ films on SiO_2_ for supported-Pt catalysts. Int. J. Green Energy.

[25] Lin C., Foucher A.C., Stach E.A., Gorte R.J. (2020). A Thermodynamic Investigation of Ni on Thin-Film Titanates (ATiO_3_). Inorganics.

[26] Bretos I., Jimenez R., Ricote J., Calzada M.L. (2018). Low-temperature crystallization of solution-derived metal oxide thin films assisted by chemical processes. Chem. Soc. Rev..

[27] Kwok C.K., Desu S.B. (1993). Low temperature perovskite formation of lead zirconate titanate thin films by a seeding process. J. Mater. Res..

[28] Huang Z., Zhang Q., Whatmore R. The role of an intermetallic phase on the crystallisation of sol-gel prepared lead zirconate titanate thin films. Proceedings of the ISAF 1998, Eleventh IEEE International Symposium on Applications of Ferroelectrics (Cat. No. 98CH36245).

