# Supplementary material for: Two-Dimensional Perovskite Crystals Formed by Atomic Layer Deposition of CaTiO3 on γ-Al2O3
Source: Nanomaterials (Basel). 2021 Aug 27;11(9):2207. doi: 10.3390/nano11092207 (PMC8469333; doi:10.3390/nano11092207)
Supplement: Supplementary file 1 [file nanomaterials-11-02207-s001.zip › nanomaterials-1348056-supplementary.pdf]

nanomaterials

Supplementary Materials

Two-Dimensional Perovskite Crystals Formed by Atomic Layer Deposition of $CaTiO_3$ on γ-Al_2O_3

Tianyu Cao, Ohhun Kwon, Chao Lin, John M. Vohs and Raymond J. Gorte *

Department of Chemical and Biomolecular Engineering, University of Pennsylvania, Philadelphia, PA 19104, USA; caot@seas.upenn.edu (T.C.); ohhun@seas.upenn.edu (O.K.); linchao@seas.upenn.edu (C.L.); vohs@seas.upenn.edu (J.M.V.)
* Correspondence: gorte@seas.upenn.edu

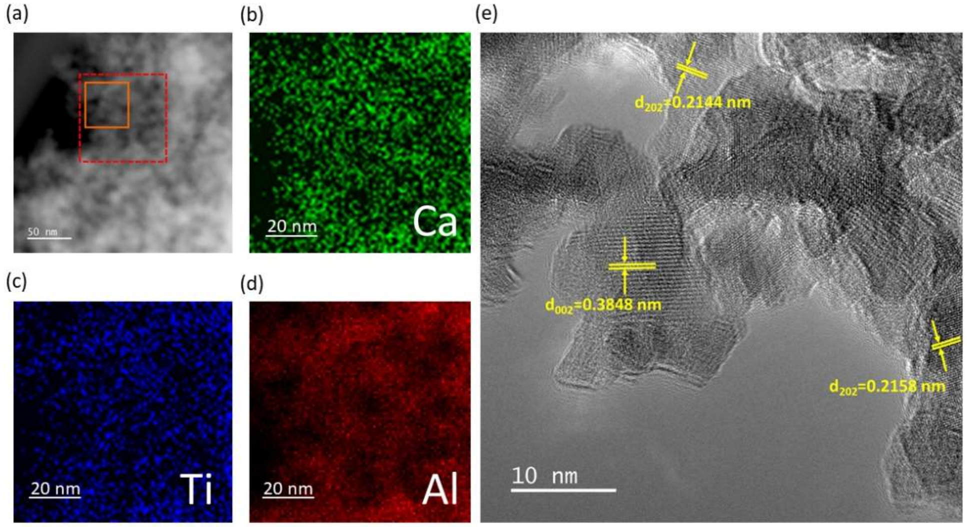

Figure S1. (a)High angle annular dark field (HAADF) STEM image of the 18 wt.% $CaTiO_3/Al_2O_3$ sample; (b), (c) and (d) are EDS maps of Ca, Ti and Al, taken from the region indicated by the dashed red frame; (e)HR-TEM of the sample, image acquired from the orange framed region.

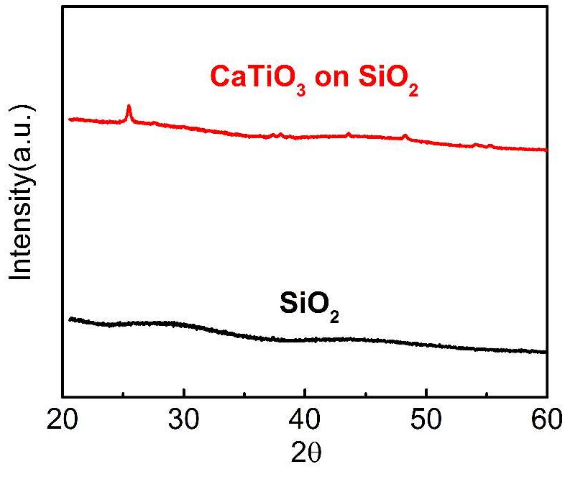

Figure S2. XRD patterns of $CaTiO_3$ deposited on SiO_2, the black line denotes bare SiO_2 and red line denotes $CaTiO_3/SiO_2$.

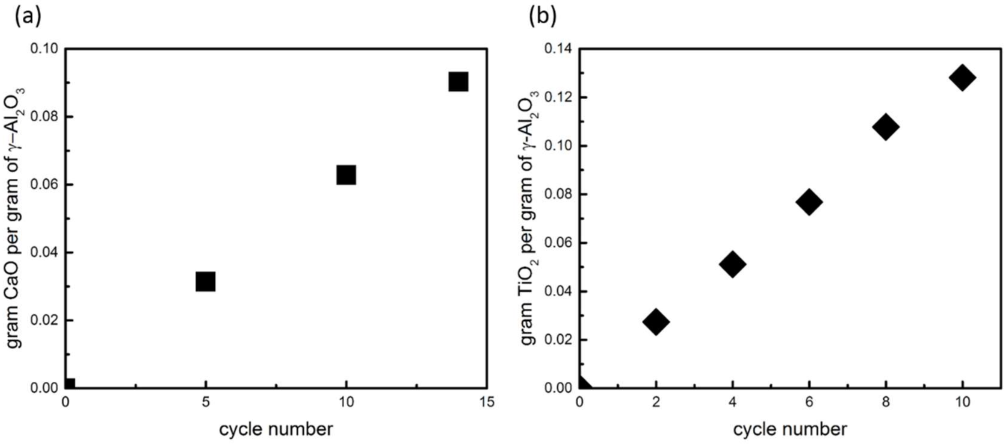

Figure S3. Growth rate of CaO, TiO_2 on γ-Al_2O_3 as a function of ALD cycles. (a)CaO; (b)TiO_2.

Preparation of $CaTiO_3$ film on the surface of SiO_2

CaO and TiO_2 were deposited to SiO_2 support with ALD. Deposition temperature as well as the precursors used are the same with that has been described in the case of $CaTiO_3/Al_2O_3$. The silica used was Q-10 SiO_2 powder (CARiACT, Fuji Silysia Chemical Ltd. Greenville, NC, USA). It was calcined in air at 1173 K for 24 h to stabilize. After this thermal treatment, specific area of the SiO_2 was 180 m^2/g.

Growth rate of Ca on the surface of SiO_2 was measured to be 5.2×10^{13} atom/cm^2 cycle, and that for Ti was 7.2×10^{13} /cm^2 cycle. After every six cycles of CaO, we deposited four cycles of TiO_2, to achieve the 1: 1 stoichiometric ratio between Ca and Ti. Weight of the sample was tracked after each cycle. The ALD process added 0.37 g of $CaTiO_3$ to every gram of SiO_2 (the weight loading was around 27%), equivalent to a film of 0.5 nm thick. Growth rate of CaO, TiO_2 on SiO_2 as a function of ALD cycles are presented in **Error! Reference source not found.**.

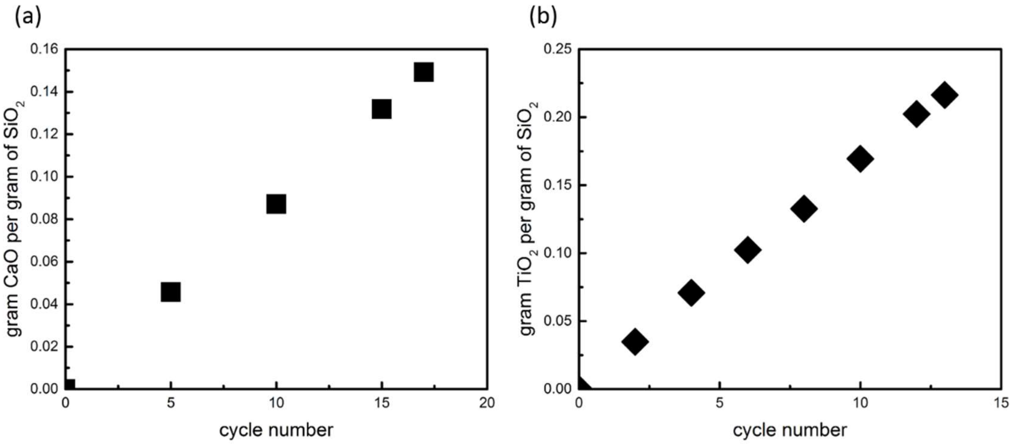

Figure S4. Growth rate of CaO, TiO_2 on SiO_2 as a function of ALD cycles. (a)CaO; (b)TiO_2.

Table S1. Lattice perimeters and d-spacing values of standard $CaTiO_3$ crystal system.

**$CaTiO_3$, orthorhombic, a=0.5386; b=0.5432; c=0.7610 (nm)**	
**(hkl)**	d-spacing (nm)
(101)	0.4396
(020)	0.2716
(200)	0.2692
(121)	0.2311
(002)	0.3805
(202)	0.2198
(040)	0.1358
(321)	0.1470
(240)	0.1213
(042)	0.1279
(123)	0.1753
